# Effects of Early or Overexpression of the *Autographa californica* Multiple Nucleopolyhedrovirus *orf94* (ODV-*e25*) on Virus Replication

**DOI:** 10.1371/journal.pone.0065635

**Published:** 2013-06-18

**Authors:** Xiao-Chun Luo, Shan-Shan Wang, Jie Zhang, Duo-Duo Qian, Si-Min Wang, Lu-Lin Li

**Affiliations:** College of Life Sciences, Central China Normal University, Wuhan, China; Wuhan Bioengineering Institute, China

## Abstract

*odv-e25*(*e25*) is one of the core genes of baculoviruses. To investigate how it functions in the replication cycle of a baculovirus, a number of *Autographa californica* multiple nucleopolyhedrovirus recombinants with *e25* under control of the promoter of immediate early gene *ie1*, or the promoter of the very late hyperexpressed gene *p10*, were constructed using a bacmid system, and the effects of early expression or overexpression of *e25* on replication of the virus were evaluated. Microscopy and titration assays demonstrated that bacmids with *e25* under control of *ie1* promoter were unable to produce budded viruses; and that the recombinant viruses with *e25* under control of *p10* promoter generated budded virus normally, but formation of occlusion bodies were dramatically reduced and delayed in the infected cells. Electron microscopy showed that there were no mature virions or intact nucleocapsids present in the cells transfected with a recombinant bacmid with *e25* under control of *ie1* promoter. Quantitative real-time PCR analysis demonstrated that alteration of the *e25* promoter did not affect viral DNA synthesis. The reporter gene expression from the promoter of the major capsid protein gene *vp39* was reduced 63% by early expression of *e25*. Confocal microscopy revealed that E25 was predominantly localized in nuclei by 24 hours post infection with wild-type virus, but it remained in the cytoplasm in the cells transfected with a recombinant bacmid with *e25* under control of the *ie1* promoter, suggesting that the transport of E25 into nuclei was regulated in a specific and strict time dependent manner.

## Introduction

*Autographa californica* multiple nucleopolyhedrovirus (AcMNPV) belongs to the Baculoviridae. During the infection cycle, AcMNPV produces two types of virions: budded virus (BV) and occlusion derived virus (ODV), which are distinct in structure and function, and are responsible for the initiation of systematic infection within the body of a host insect and to spread infection to different members of susceptible insect species, respectively [1]. Both BV and ODV contain enveloped rod-shaped nucleocapsids that are assembled in the nucleus. In the early phase of infection, newly assembled nucleocapsids exit the nucleus and acquire an envelope by budding through the plasma membrane that is pre-modified by viral proteins, producing mature BVs. After budding, BVs attach to other susceptible cells to initiate secondary infections [2], [3]. In the late phase, nucleocapsids are enveloped by viral induced membranes within the nucleoplasm, forming ODVs, which are occluded in a protein crystal matrix, named occlusion bodies (OBs). Upon lysis of the infected cells, OBs are released into the environment. When OBs are consumed by another susceptible insect, the ODV virions are released to infect the midgut epithelial cells, initiating a new infection cycle [4]. BV and ODV share the same genome sequence, but differ in the composition of proteins associated with the envelope. The BV envelope contains several virally encoded proteins including GP64 that is a low pH activated envelope fusion protein that is required for entry of BV into cells [5], [6]. In contrast, many of the ODV envelope-associated proteins differ from BV. ODV contains a group of proteins named *per os* infectivity factors required for oral infection and several other proteins [7]–[14]. In *Helicoverpa armigera* NPV (HearNPV), there are 12 BV-specific and 21 ODV-specific envelope proteins identified by comprehensive proteomics analyses [15].

ODV-E25 (E25) was originally identified as a 25 KD protein in *Orgyia pseudotsugata* MNPV (OpMNPV) and AcMNPV, and was localized to the envelopes of ODV in OpMNPV [13]. Proteome analyses have shown that E25 is an ODV component in AcMNPV, HearNPV and *Chrysodeixis chalcites* NPV, and also a component of BV in AcMNPV [14], [16]–[18]. E25 of OpMNPV, AcMNPV and *Spodoptera litura* MNPV was detected in infected cells as doublets of about 25–26 KD and 27–28 KD, respectively [13], [19]. AcMNPV E25 and several additional envelope proteins contain an N-terminal hydrophobic sequence in combination with several adjacent positively charged amino acids, which are predicted to be motifs that target these proteins to the nuclear envelope, intranuclear microvesicles and ODV envelopes [7], [20]. The intranuclear microvesicles are thought to be precursors from which the envelopes of ODVs are derived. In AcMNPV, E25 is encoded by *orf94*, which has orthologs in the genomes of all baculoviruses sequenced to date and is considered a baculovirus “core gene” [21], [22]. It was recently reported that AcMNPV *e25* is required for budded virus infectivity and occlusion derived virus formation [23]. However, it is still unknown how E25 functions in viral replication.

Replication of AcMNPV and other baculoviruses proceeds through in a series of well-ordered stages, which are administrated by an expression cascade of the viral genes. The gene expression of the viruses can be divided into early, late and very late phases. Each gene has a specific time course of expression in virus replication cycle. Generally, genes encoding the proteins which are involved in viral DNA replication and/or late gene expression (eg. *dnapol*, *lef1-12*) are expressed at an early time; structural protein genes (eg. *vp39*, *p6.9* and *e25*) are expressed at late times; some genes are expressed at both early and late phases (eg. *ie1*, *pp31* and *gp64*); *p10* and *polyhedrin* (*polh*) are highly expressed very late genes, which are expressed through late and very late times of infection [24]. Although many reports have described investigations of the effects of baculovirus gene knockouts on the virus life cycle, few have examined the effects of altering the temporal or elevated expression of genes. The altered temporal expression of an essential core gene, such as *e25*, could have an impact on virus replication by disrupting the normal progression of molecular events. Hence, it is possible to investigate the role of a viral gene from phenotypic variations induced by temporal changes in its expression. In this study, the effects of early expression or very late overexpression of *e25* on the replication of AcMNPV was investigated. It was found that early expression of *e25* severely disrupted both BV and ODV production. Although the overexpression of *e25* did not have significant effects on BV production or assembly of virions, it inhibited the formation of occlusion bodies.

## Results

### Generation of recombinant AcMNPV bacmids with *e25* under control of alternative promoters

**Figure 1:**
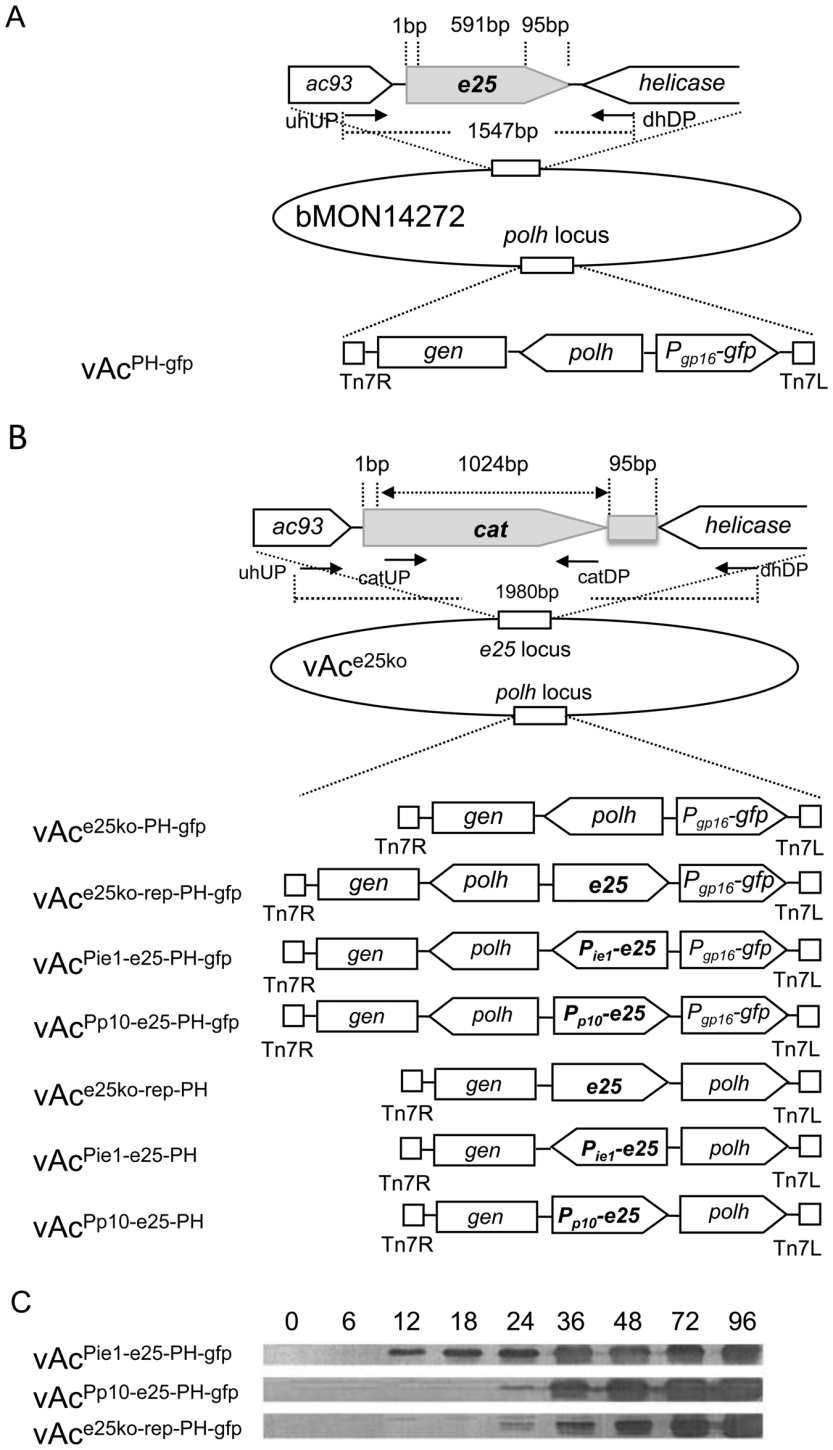
Construction of recombinant AcMNPV bacmids. (A) Schematic map of the structures of the *orf94* (*e25*) locus and the *polh* locus in a wt AcMNPV bacmid vAc^PH-gfp^. A copy of *egfp* ORF under control of AcMNPV *gp16* promoter and a copy of *polh* were inserted into the *polh* locus in opposite orientation. (B) Schematic maps of the structures of the *e25* locus and the *polh* locus in *e25* knockout and repair bacmids. In *e25* locus, a 591 bp sequence of the *orf94* was deleted and replaced with the *cat*. In *polh* locus, a copy of *egfp* ORF under control of the *gp16* promoter and a *polh* were inserted into the *polh* locus in opposite orientation (vAc^e25ko-PH-gfp^), or an *e25* with native promoter (vAc^e25ko-rep-PH-gfp^) or *ie1* promoter (vAc^Pie1-e25ko-PH-gfp^) or *p10* promoter (vAc^Pp10-e25ko-PH-gfp^) was additionally inserted between *polh* and *egfp*. Alternatively, a *polh* with native promoter and an *e25* with native promoter (vAc^e25ko-rep-PH^) or *ie1* promoter (vAc^Pie1-e25ko-PH^) or *p10* promoter (vAc^Pp10-e25ko-PH^) was inserted in the *polh* locus in opposite orientation. (C) Time course analysis of the E25 expressed in Sf9 cells infected by vAc^Pie1-e25-PH-gfp^, vAc^Pp10-e25-PH-gfp^ or vAc^e25ko-rep-PH-gfp^. The cells transfected with the individual bacmids were harvested at designated time points post transfection, and the cell extracts subjected to SDS-PAGE, and immunoblot analysis with E25-specific antiserum.

To determine the effect of the changes in the time course of expression of *e25* on virus replication, several recombinant bacmids were constructed, in which the original *orf94* was deleted and another copy of *orf94* with an alternative promoter was inserted back into the bacmids at the *polh* locus. At first, an *e25* knockout bacmid, vAc^e25ko^, was constructed, in which the 5′-end of the *e25* ORF (nt2-592) was deleted and replaced with the chloramphenicol acetyltransferase (*cat*) gene facilitating antibiotic selection in *E. coli* (Fig. 1A & B). Two bacmids designed to express E25 in early phase or over-express the protein in very late phase in virus replication cycle and an *e25*-knockout repair bacmid were constructed by inserting an *orf94* under control of AcMNPV *ie1* (vAc^Pie1-e25-PH-gfp^), *p10* (vAc^Pp10-e25-PH-gfp^), or its native promoter (vAc^e25ko-rep-PH-gfp^) into vAc^e25ko^ at the *polh* locus. A copy of the AcMNPV *polh* and the reporter gene *egfp* (enhanced green fluorescence protein) was also placed at the same locus in the bacmids (Fig. 1B). AcMNPV *ie1* is an immediate early gene, which is expressed early and continues to be expressed through the late phase in infection [25], [26]. *p10* is a highly expressed very late gene. A time course analysis of E25 production in the cells transfected with vAc^Pie1-e25-PH-gfp^, vAc^Pp10-e25-PH-gfp^, or vAc^e25ko-rep-PH-gfp^ was performed by western-blots of extracts of the transfected cells with the E25-specific antiserum. As shown in Fig. 1C, the E25 protein was detected as early as 12 hours post transfection (h.p.t.) in extracts of the cells transfected by vAc^Pie1-e25-PH-gfp^. In contrast, it was not detected until 24 h.p.t. in the cells transfected by two other bacmids. This proved that E25 was expressed early in cells transfected with vAc^Pie1-e25-PH-gfp^, driven by the *ie1* promoter.

Two additional bacmids vAc^PH-gfp^ (Fig. 1A) and vAc^e25ko-PH-gfp^ (Fig. 1B) were constructed by inserting a copy of the *polh* and *egfp* into the AcMNPV bacmid bMON14272 or vAc^e25ko^. They were respectively used as wild-type (wt) and *e25*-negative controls in this study.

### Effects of early/over expression of the *e25* on virus production

**Figure 2:**
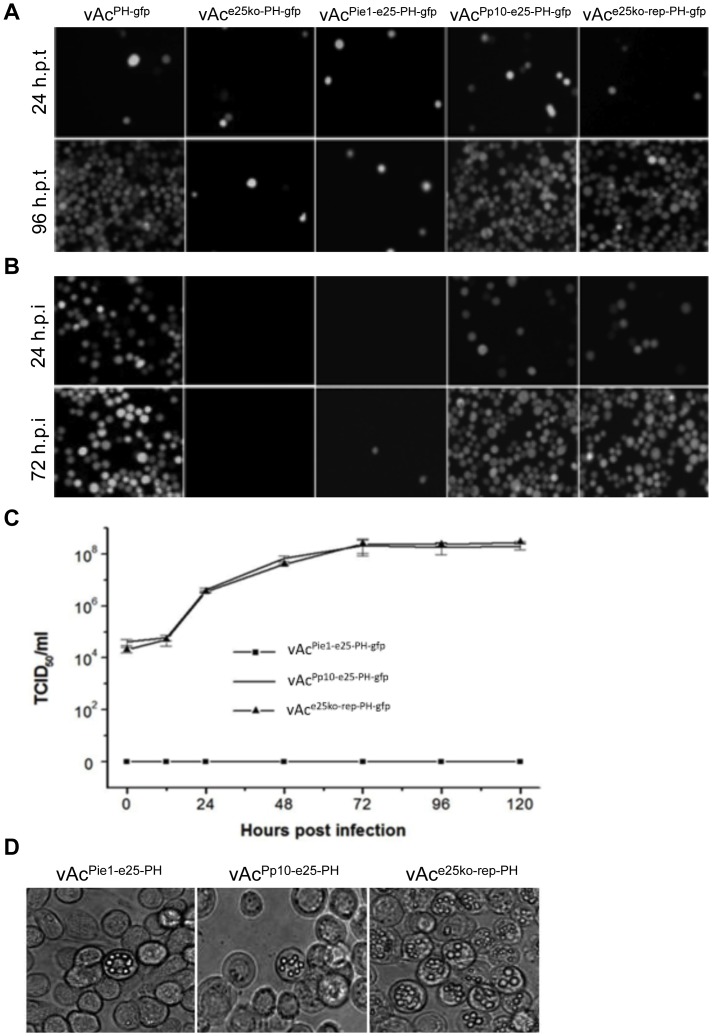
Analysis of viral replication in Sf9 cells transfected/infected with AcMNPV recombinants with *e25* under control of alternative promoters. (A) Fluorescence microscopy of Sf9 cells transfected with vAc^PH-gfp^, vAc^e25ko-PH-gfp^, vAc^Pie1-e25-PH-gfp^, vAc^Pp10-e25-PH-gfp^ or vAc^e25ko-rep-PH-gfp^, at 24 and 96 h.p.t. (B) Fluorescence microscopy of Sf9 cells infected with the supernatants from transfections above, at 24 and 72 h.p.i. (C) Virus growth curves of vAc^Pie1-e25-PH-gfp^, vAc^Pp10-e25-PH-gfp^ and vAc^e25ko-rep-PH-gfp^ in Sf9 cells. Sf9 cells were inoculated with the supernatant from the cell cultures transfected by vAc^Pp10-e25-PH-gfp^ or vAc^e25ko-rep-PH-gfp^ at a MOI of 5, or 1000 µl of the supernatant from the cell culture transfected by vAc^Pie-e25-PH-gfp^ and harvested at the designated time points, and virus titers were determined by TCID_50_ end-point dilution assays. Each data point represents the average titer of three independent infections. Error bars indicate standard deviations. (D) Phase-contrast microscopy of Sf9 cells transfected with vAc^Pie-e25-PH^ (96 h.p.t.), vAc^Pp10-e25-PH^ (120 h.p.t.) or vAc^e25ko-rep-PH^ (96 h.p.t.).

To examine the effects of early expression or overexpression of the *e25* gene on virus replication, the bacmids vAc^Pie1-e25-PH-gfp^ and vAc^Pp10-e25-PH-gfp^ were separately transfected into Sf9 cells. vAc^PH-gfp^, vAc^e25ko-rep-PH-gfp^ and vAc^e25ko-PH-gfp^ were used as controls. Cells were then incubated with supernatants from transfected cell cultures and monitored by fluorescence and phase contrast microscope. As shown in Fig. 2A, fluorescence was first observed in cells transfected with all the five individual bacmids, at 24 h.p.t. No obvious difference in number and lightness of the fluorescent cells was observed. At 96 h.p.t., the majority of the cells in the dishes containing vAc^PH-gfp^, vAc^Pp10-e25-PH-gfp^, or vAc^e25ko-rep-PH-gfp^ were fluorescent, whereas there was no significant increase in the number of fluorescent cells with vAc^Pie1-e25-PH-gfp^ or vAc^e25ko-PH-gfp^, suggesting that the spread of infection occurred in the cells with wt, *e25*-knockout repair, or the bacmid with *e25* under control of the *p10* promoter; but not in the cells transfected with the bacmid with *e25* deleted or the one with *e25* driven by the *ie1* promoter.

At 120 h.p.t., supernatants were collected from the individual transfections and added to newly plated Sf9 cells. Fluorescence was first observed in the cell cultures inoculated with the supernatants with vAc^PH-gfp^, vAc^Pp10-e25-PH-gfp^, or vAc^e25ko-rep-PH-gfp^, at 24 hours post infection (h.p.i.). And almost all cells in these dishes were filled with fluorescence by 72 h.p.i. In contrast, no fluorescent cells were observed in the dish inoculated with the supernatant from the transfection with vAc^e25ko-PH-gfp^, up to 72 h.p.i. A few fluorescent cells were occasionally observed in the dish inoculated with supernatant from the transfection with vAc^Pie1-e25-PH-gfp^ (Fig. 2B). These observations indicated that the wt and the *e25*-knockout repair bacmid as well as the bacmid with *e25* under control of the *p10* promoter replicated in the transfected cells and produced infectious viruses, whereas virus replication was severely disrupted in the cells transfected with the *e25* knockout bacmid or the one with *e25* under control of the *ie1* promoter.

The effects of early or late overexpression of *e25* on virus replication were further examined by a virus growth curve analyses. As shown in Fig. 2C. the virus titer from the cell culture transfected with vAc^Pie1-e25-PH-gfp^ cells was too low to detect at all time points up to120 h.p.i., whereas Sf9 cells infected with vAc^Pp10-e25-PH-gfp^ revealed a steady increase in virus production, similar to the cells infected with vAc^e25ko-rep-PH-gfp^. These results indicate that early expression of the *e25* blocks production of infectious budded virus, whereas overexpression of the gene driven by the *p10* promoter does not have significant effects on the BV replication of the AcMNPV.

Under phase contrast microscopy, OBs were seen in the cells transfected individually with vAc^PH-gfp^, vAc^e25ko-rep-PH-gfp^, vAc^Pie1-e25-PH-gfp^ and vAc^e25ko-PH-gfp^ at late times, but few OB-containing cells and no evidence of the spreading of the infection was observed in the transfections with vAc^Pie1-e25-PH-gfp^ or vAc^e25ko-PH-gfp^ (data not shown). To eliminate potential effects from the extra *gp16* promoter and the *egfp* sequence inserted at the same locus, three additional bacmids vAc^e25ko-rep-PH^, vAc^Pie1-e25-PH^ and vAc^Pp10-e25-PH^ were constructed (Fig. 1B). OBs were first observed at 48 h.p.t. in the cells transfected with vAc^e25ko-rep-PH^ and the cells with vAc^Pie1-e25-PH^. By 96 h.p.t., the majority of cells transfected by vAc^e25ko-rep-PH^ were filled with OBs. In contrast, OBs were observed in only a few isolated cells in the cultures transfected with vAc^Pie1-e25-PH^. In the cells transfected with vAc^Pp10-e25-PH^, OBs were occasionally found in few cells by 120 h.p.t. (Fig. 2D).

### Effects of early expression or late overexpression of *e25* on virus morphogenesis

**Figure 3:**
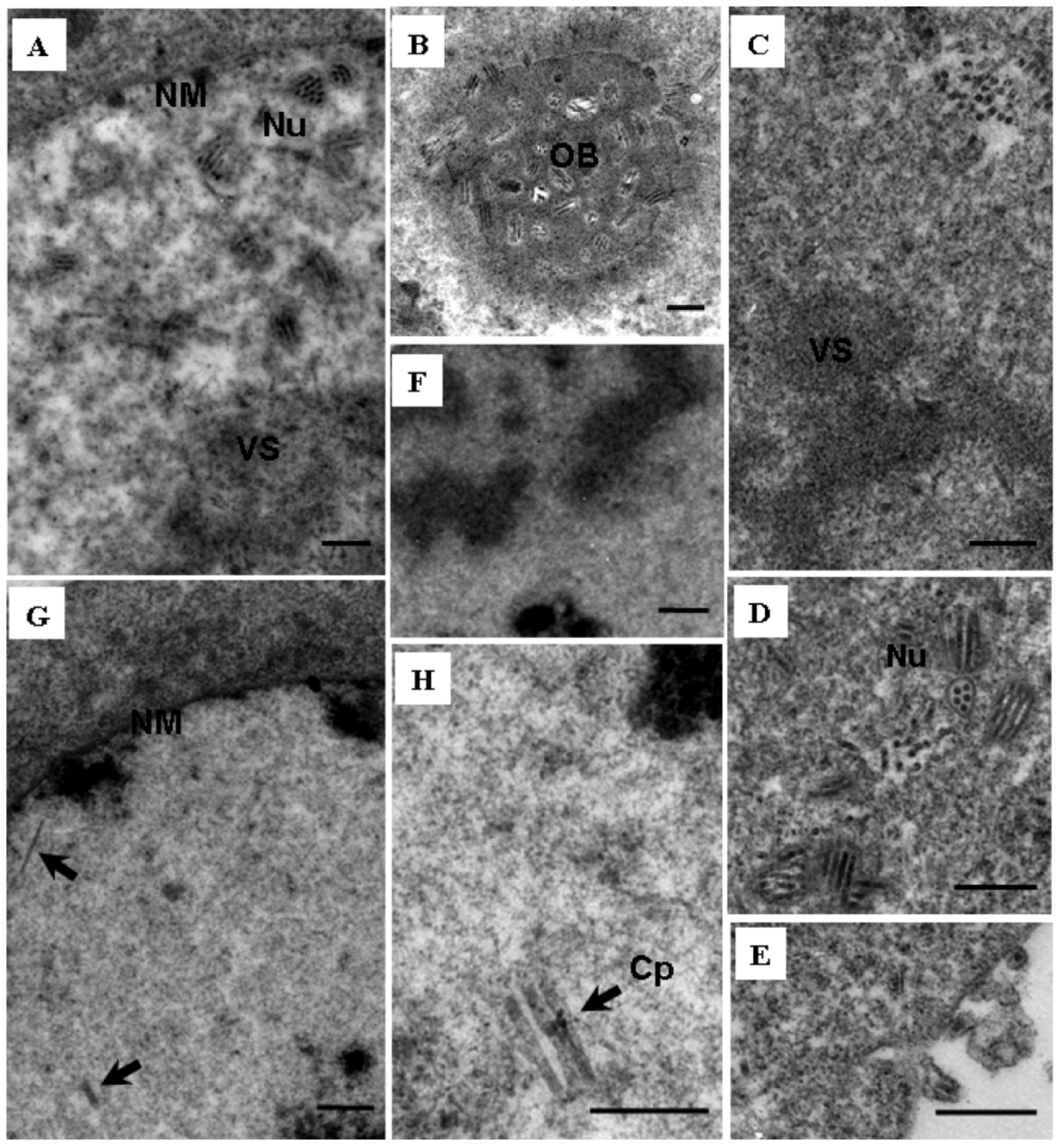
Transmission electron microscopy analysis of Sf9 cells transfected with vAc^e25ko-rep-PH-gfp^ (A and B), vAc^Pp10-e25-PH-gfp^ (C-E), or vAc^Pie-e25-PH-gfp^ (F-H), at 96 h. **p.t.** (A) Nucleocapsids (Nu) present around the virogenic stroma in an enlarged nucleus (VS). (B) Virions embedded in an occlusion body (OB). (C) Virogenic stroma with a few nucleocapsids associated. (D) Enveloped nucleocapsids. (E) Virions budding through cytoplasmic membrane. (F) Virogenic stroma-like structure. (G) Two rod-shaped nucleocapsid-like particles present in an enlarged nucleus. (H) Empty rod-shaped capsids (Cp). Scale bar = 500 nm.

To further determine if temporal alteration in expression of *e25* had any effect on virus morphogenesis, electron microscopic analysis was performed with thin sections of the cells transfected with vAc^Pie1-e25-PH^, vAc^Pp10-e25-PH^ or vAc^e25ko-rep-PH^. At 96 h.p.t., the cells transfected with vAc^e25ko-rep-PH^ showed the typical characteristics of a baculovirus infection. Virogenic stroma inundated with rod-shaped nucleocapsids (Fig. 3A) and nucleocapsids acquiring their envelopes and embedding into the developing OBs (Fig. 3B) were observed. Similarly, virogenic stroma (Fig. 3C), abundant enveloped nucleocapsids (Fig. 3D) within enlarged nuclei and single nucleocapsids budding through cytoplasmic membrane could also be observed (Fig. 3E) in the cells transfected with vAc^Pp10-e25-PH^, but OBs were not found, obviously due to their rareness (as shown in Fig. 2D). In the cells transfected with vAc^Pie1-e25-PH^, virogenic stroma-like structures could be observed (Fig. 3F), but there were not any mature nucleocapsids present. Only a few rod-shaped empty capsids were observed in the nuclei (Fig. 3G & H). The electron microscopy indicated that whereas early expression of *e25* interfered with nucleocapsid assembly, late overexpression of *e25* had no effect on nucleocapsid assembly, but interfered with OB formation.

### Effects of deletion or early expression of the *e25* on virus DNA replication

**Figure 4:**
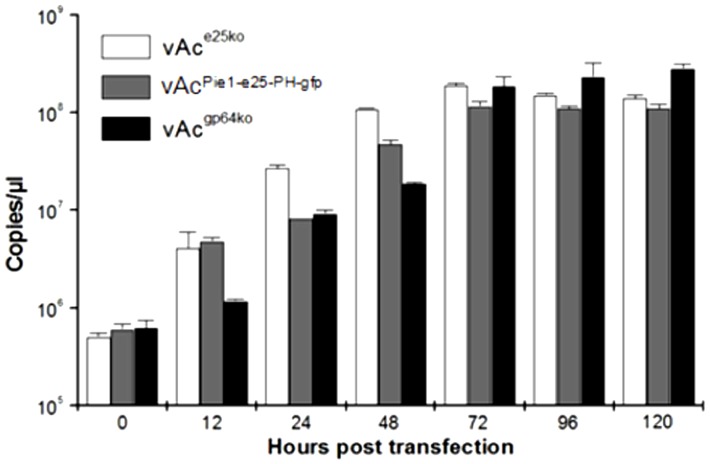
Quantitative-PCR analysis of viral DNA replication in Sf9 cells transfected by recombinant AcMNPV bacmids with *e25* deleted or under control of *ie1* promoter. Total DNA was purified from the cells transfected with vAc^e25ko^, vAc^Pie1-e25-PH-gfp^, or vAc^gp64ko^, at 0, 12, 24, 48, 72, 96 and 120 h.p.t., digested with DpnI to eliminate input bacmid DNA, and analyzed by real-time PCR. The values displayed represent the averages from transfections performed in triplicate with error bars indicating standard deviations.

The levels of viral DNA replication in the cells transfected individually with vAc^e25ko^ and vAc^Pie1-e25-PH-gfp^ were measured over a 120 h time-course, to determine if *e25* had an impact on viral DNA replication. The transfected Sf9 cells were collected at designated time-points, and the total DNA was extracted and analyzed by qPCR. vAc^gp64ko^, which is a *gp64*-knockout mutant of the wild type bacmid bMON14272 was used as a control. For all three viruses, DNA synthesis began increasing at 12 h.p.t. and continued until 72 h.p.t. (Fig. 4). The levels of DNA detected for vAc^e25ko^ were higher than for vAc^Pie1-e25-PH-gfp^ and vAc^gp64ko^ before 72 h.p.t. The levels of vAc^Pie1-e25-PH-gfp^ were higher than vAc^gp64ko^ at 12 h.p.t. and 48 h.p.t. However, the peak levels reached by all the three bacmids at 72 h.p.t. were similar. These results indicated that the total level of replication in individual infected cells was unaffected by deletion or early expression of *e25*, although the DNA replication might be accelerated slightly by the mutations. This could also be due to the lack of BV production, which would cause the DNA to accumulate in the KO, and P_ie1_-e25 cells.

### Early expression of the *e25* knocks down *gus* expression driven by *vp39* promoter

Effects of early expression of the *e25* on virus gene expression were also evaluated by assays using a β-glucuronidase (GUS) gene under control of the *vp39* promoter. *vp39* encodes the major capsid protein. It is expressed in late phase in infection [27].

**Figure 5:**
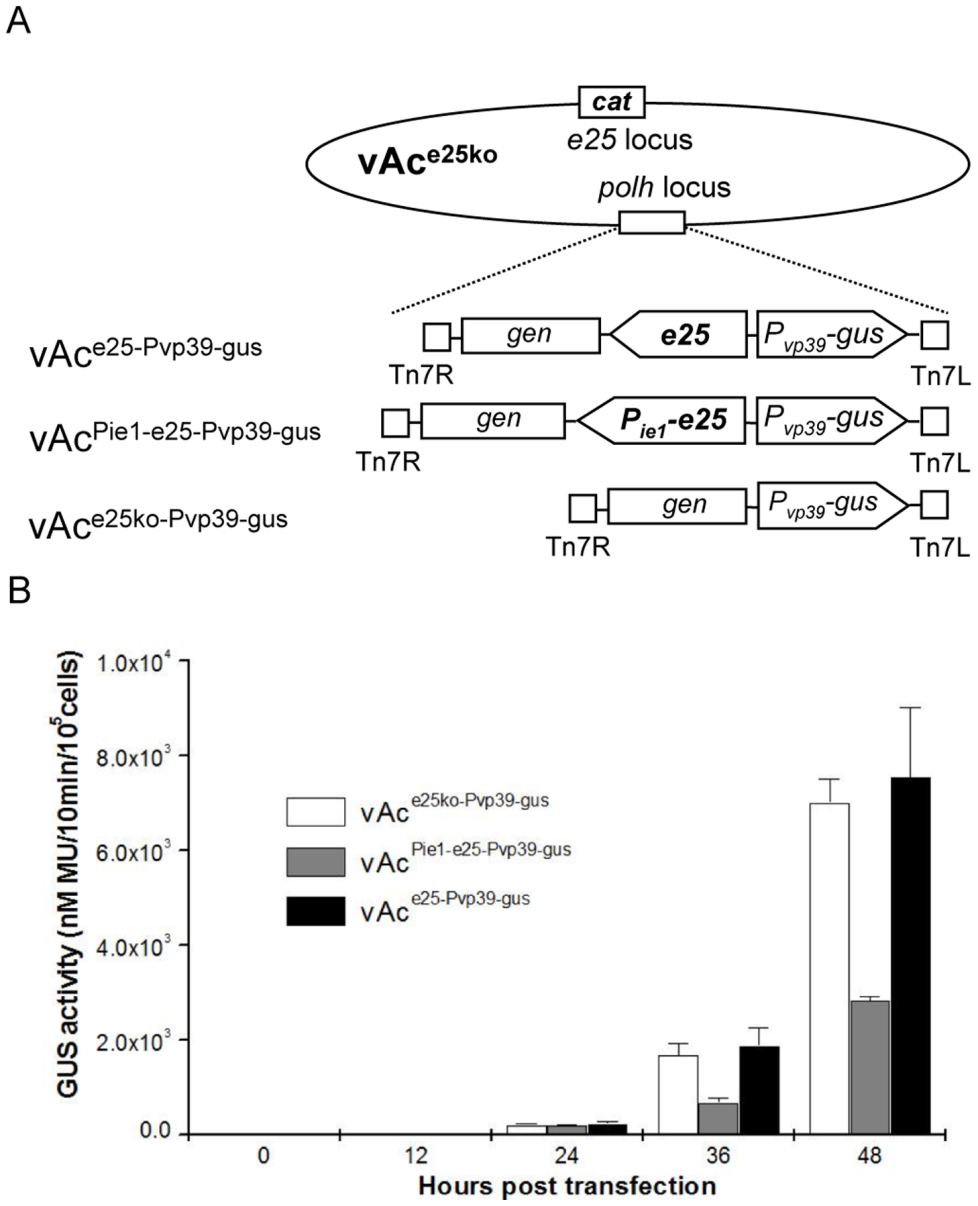
Analysis of late gene expression in Sf9 cells transfected by recombinant AcMNPV bacmids with *e25* deleted or under control of alternative promoters. (A) Schematic maps of the structures of the recombinant AcMNPV bacmids with *e25* mutants. A copy of *gus* under control of *vp39* promoter alone (vAc^e25ko-Pvp39-gus^), or linked with a copy of *e25* with native promoter (vAc^e25-Pvp39-gus^) or *ie1* promoter (vAc^Pie1-e25-Pvp39-gus^) in opposite orientation, was inserted into the *polh* locus of vAc^e25ko^. (B) GUS assays of the extracts of the Sf9 cells transfected with the vAc^e25ko-Pvp39-gus^, vAc^e25-Pvp39-gus^ or vAc^Pie1-e25-Pvp39-gus^. Shown are the levels of GUS activity detected in the transfected cells. GUS activity is expressed as nanomoles of 7-hydroxy-4 methycoumarin (MU) produced from 4-methylumbelliferyl β-D-glucuronide by β-glucuronidase expressed in 10^5^ transfected cells.

Three late expression reporter bacmids vAc^e25ko-Pvp39-gus^, vAc^Pie1-e25-Pvp39-gus^ and vAc^e25-Pvp39-gus^, which contain the *gus* under control of a *vp39* promoter, were constructed (Fig. 5A). All of the reporter bacmids have the original *e25* deleted. vAc^Pie1-e25-Pvp39-gus^ and vAc^e25-Pvp39-gus^ have a copy of *e25* under control of an *ie1* promoter and an *e25* with the native promoter inserted at the *polh* locus respectively. Sf9 cells transfected with individual reporter bacmids were collected at designated time points and used for GUS assays.

GUS activity was first detected at 24 h.p.t. in all cases. The levels of GUS activity detected in extracts of the cells transfected with vAc^e25ko-Pvp39-gus^, were similar to the ones of the cells transfected with vAc^e25-Pvp39-gus^ at all time points up to 48 h.p.t. However, GUS activity was significantly lower in the cells transfected with vAc^Pie1-e25-Pvp39-gus^ than the ones in the cells transfected with the two other reporter bacmids, being 64% and 63% lower than the GUS activities in the cells transfected with vAc^e25-Pvp39-gus^, at 36 and 48 h.p.t. respectively (Fig. 5B). These results suggest that deletion of *e25* has minimum effects on, but early expression of *e25*, reduces late gene expression driven by the *vp39* promoter.

### Localization of E25 in AcMNPV-infected insect cells

Subcellular localization of E25 in infected Sf9 cells was analyzed by immunofluorescence microscopy in combination with nuclear staining by Hoechst33258. Sf9 cells were infected with vAc^PH-gfp^ at a MOI of 5. At designated time points, the cells were sampled, blotted with E25-specific antiserum and Rhodamine-conjugated goat-anti-rabbit IgG, stained with Hoechst33258, and subjected to confocal microscopy.

**Figure 6:**
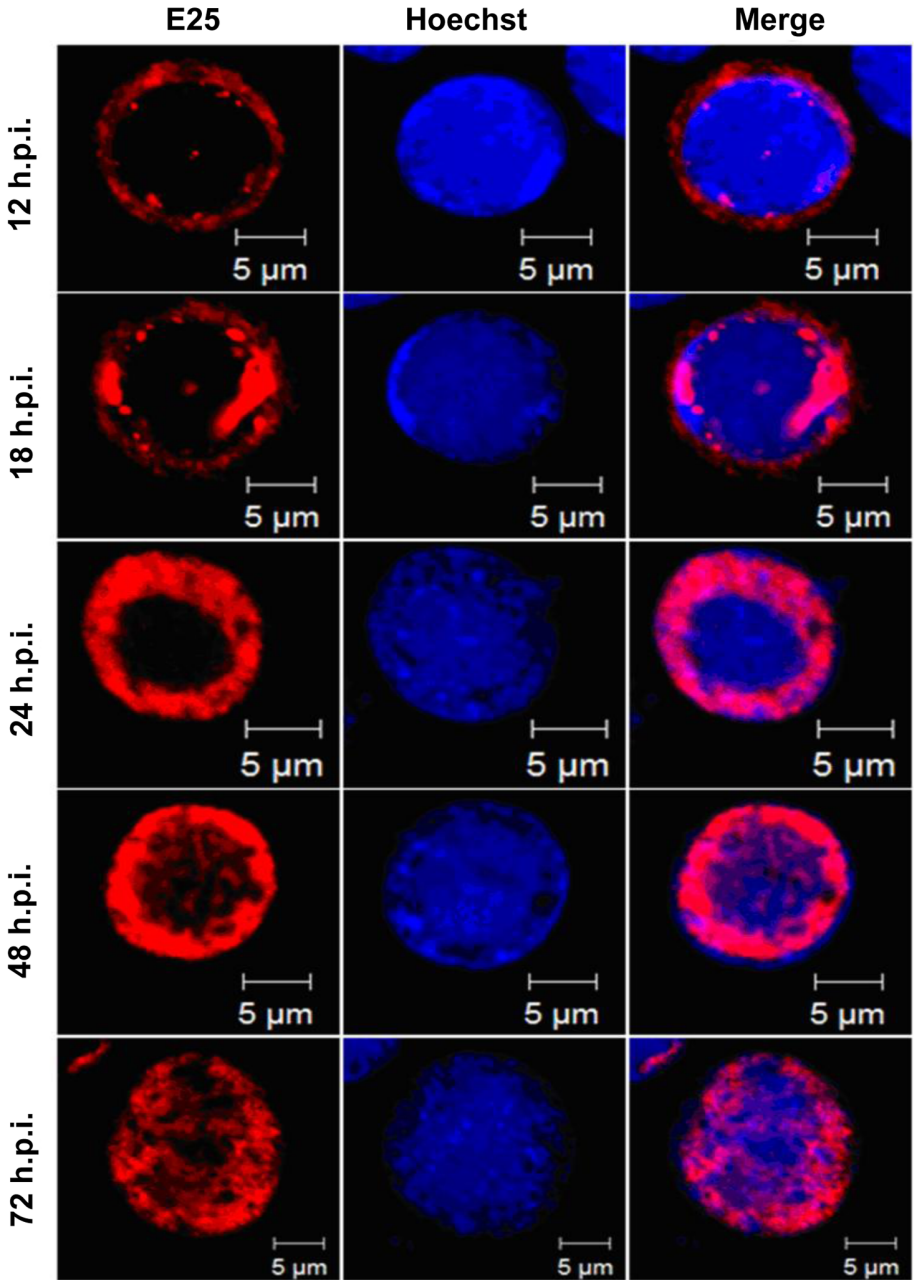
Subcellular localization of the E25 in Sf9 cells infected by AcMNPV. Sf9 cells infected by AcMNPV were sampled at 12, 18, 24, 48 and 72 h.p.i., blotted with E25-specific polyclonal antibodies which were subsequently blotted by using Rhodamine-conjugated goat-anti-rabbit IgG to label E25 (red), stained with Hoechst33258 to mark nuclei (blue), and subjected to confocal microscopy.

The E25 labeled by Rhodamine with red fluorescence was first observed predominantly in the cytoplasm at 12 h.p.i. (Fig. 6). At 18 h.p.i., about half of the red fluorescence was present in nuclei (blue color) in dot like structures. By 24 h.p.i., red fluorescence could only be observed in nuclei, forming a ring zone at periphery of the nucleus. By 72 h.p.i., red fluorescence spread throughout the nucleus (Fig. 6).

### Early expression of *e25* blocks nuclear transporting of E25

Localization of E25 in the Sf9 cells transfected by vAc^Pie1-e25-PH-gfp^ was analyzed by immunofluorescence microscopy in the similar way as mentioned above.

**Figure 7:**
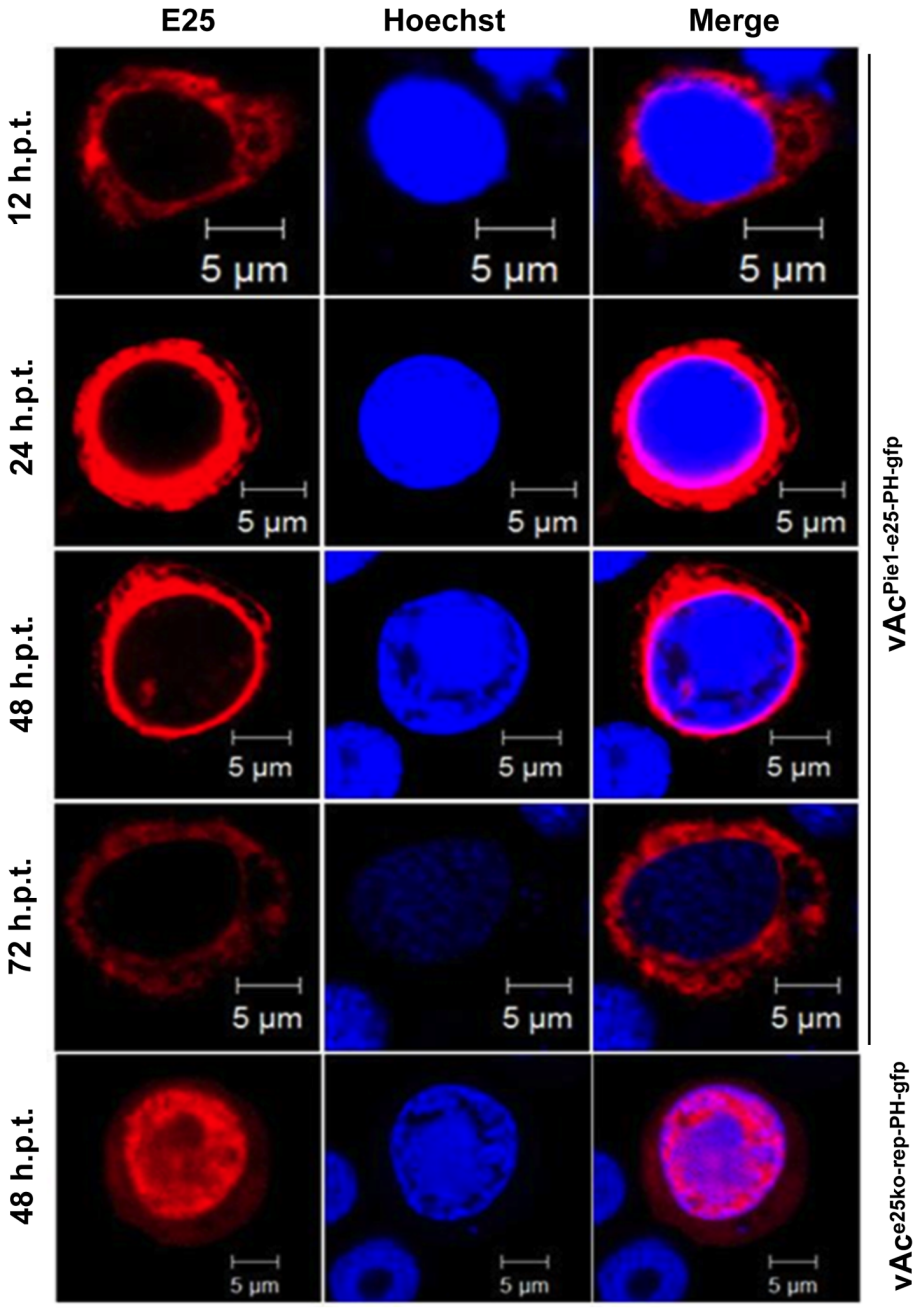
Subcelluar localization of E25 in Sf9 cells infected by AcMNPV mutant with *e25* under control of early gene promoter. The cells transfected by vAc^Pie1-e25-PH-gfp^ were sampled at 12, 24, 48 and 72 h.p.t., blotted with E25-specific polyclonal antibodies which were subsequently blotted by using Rhodamine-conjugated goat-anti-rabbit IgG to label E25 (red), stained with Hoechst33258 to mark nuclei (blue), and subjected to confocal microscopy. Sf9 cells transfected by vAc^e25ko-rep-PH-gfp^, which were sampled at 48 h.p.t. and treated in the same way, were shown as control.

The E25 expressed by *ie1* promoter was observed first in the cytoplasm at 12 h.p.t., as red fluorescence emitted by Rhodamine-conjugated goat-anti-rabbit IgG, which was associated with E25 through E25-specific antibodies (Fig. 7). No red fluorescence signal was found in the nuclei until 24 h.p.t. At 48 h.p.t., a small region of red fluorescence was observed in the nuclei, but the density of the red fluorescence did not increase by 72 h.p.t., most still remained in the cytoplasm. In contrast, when expressed from its own promoter, E25 was predominantly localized in the nucleus by 48 h.p.t. (Fig. 7). This phenomenon demonstrated that the early expression of E25 prevented the trafficking E25 into the nucleus in a transfected cell.

## Discussion

The infection cycle of AcMNPV and other baculoviruses is organized by a complex transcriptional cascade. Early genes are expressed prior to DNA replication and are transcribed by host cell RNA polymerase II [28], [29]. Late and very-late genes are transcribed following the onset of DNA replication by a virus-encoded RNA polymerase [28], [30]–[32]. The mechanism controlling the transition from early gene expression and DNA replication to late gene expression is not clear. *e25* is expressed at the late phase in natural replication cycles of AcMNPV and locates in the nuclei of the infected cells [13]. To determine effects of the temporal change of expression of *e25* on virus replication, AcMNPV recombinants expressing E25 under control of the promoter of the immediate early gene *ie1* or the promoter of the overexpressed very late gene *p10*, were constructed in this study; and the phenotypic variations induced by the temporal changes of expression of the *e25* were analyzed. We found that early expression of *e25* almost completely eliminated production of infectious BV (Fig. 2). In addition, in the cells transfected with the bacmids in which the *e25* was placed under control of the *ie1* promoter, no mature virions or intact nucleocapsids were observed (Fig. 3). In contrast, overexpression of *e25* did not cause significant change in the production, infectivity of BV, or assembly of virions in the nuclei of infected cells.

To explore the mechanism behind the phenotypic variations resulting from the early expression of *e25*, the effects on viral DNA replication and late gene expression were evaluated by Q-PCR and by transient assays. It was shown that early expression of *e25* did not affect viral DNA replication (Fig. 4), but resulted in a drop of 63% in expression level of the reporter gene driven by the *vp39* promoter (Fig. 5). Reduction in expression of some late genes could affect virus production. How the early expression of *e25* results in reduction of another late gene remains to be elucidated.

Since ODVs are assembled in nuclei of infected cells, all structural proteins have to translocate from the site they are synthesized in cytoplasm into nuclei. It has been shown that the ODV envelope proteins traffic through the ER, outer nuclear membrane, inner nuclear membrane, and nuclear pore complex, which is a continuous network of membranes [33]. To test if early expression of *e25* affects the transport of E25 into nuclei, the subcellular localization of E25 in the cells transfected with the bacmid with *e25* under control of *ie1* promoter were tracked by immunofluorescence assays, and compared with the cells transfected with the *e25* knockout repair bacmid. As a result, E25 in the cells transfected by vAc^Pie1-e25-PH-gfp^ mostly remained in the cytoplasm until 72 h.p.t., in contrast to the cells transfected by vAc^e25ko-rep-PH-gfp^ where E25 was almost completely localized in the nuclei by 24 h.p.t. This indicates that transport of E25 into nuclei was regulated in a time specific manner, in infected cells. E25 was previously shown to interact with ODV-E66, which also bound to FP25K and BV/ODV-E26 [34]. FP25K and BV/ODV-E26 are involved in trafficking of viral envelope proteins [33], [35]. Early expression of E25 may cause changes in interactions between E25 and other viral or host proteins or in modifications on E25, that subsequently interrupt trafficking of E25 into the nucleus. If E25 is involved in envelopment of ODV, as proposed [10], blocking of transporting E25 into nuclei would affect ODV envelopment that occurs in nuclei. This is evidenced by the observation from electron microscopy of the cells transfected with vAc^Pie1-e25-PH^, where there were no enveloped virions present. It was previously reported that there were no virions found in the cells transfected with a recombinant AcMNPV bacmid with *e25* deleted [23]. However, the early expression of E25 suggests that it is involved in the proper assembly of nucleocapsids independent of its role in the ODV envelope. The early expression of E25 could alter DNA packaging by binding to and preventing the assembly of a component of the packaging complex that it normally interacts with later in infection. This could account for the apparent lack of DNA in the nucleocapsids, although it remains to be proven by additional experimental evidence.

To date, more than twenty proteins have been identified to be associated with envelopes of ODV and/or BV in AcMNPV and other baculoviruses. E25 is among a few envelope proteins present in both ODV and BV. BVs acquire their envelopes from modified plasma membrane whereas ODVs are thought to obtain theirs from intranuclear membranes. This suggests that E25 must be partitioned between both the cytoplasm and nucleus during virus replication. Although great progress have been made in deciphering the pathway of the ODV envelope proteins from their site of insertion into the membrane of ER through their transit to the inner nuclear membrane [7], [33], [36], the mechanism directing these proteins into nuclei or onto the plasma membrane remains to be determined.

In this study, the effects of very late overexpression of *e25* on replication of AcMNPV were also evaluated. In cells infected with the recombinant virus with *e25* placed under control of the *p10* promoter, the production of BV was not affected by overexpression of *e25*, in comparison with the cells infected by the virus containing *e25* with native promoter, but OB production was limited and they were found only in a few cells in very late phase in infection (Fig. 2). This result demonstrates that redundant E25 in the nuclei may inhibit occlusion of virions and formation of OB. However, it could also result from competition from the extra *p10* promoter added at the *polh* locus. It was previously reported that competition between baculovirus *polh* and *p10* gene expression occurred during infection of insect cells [37]. In this study, the polyhedrin detected in the cells infected with vAc^Pp10-e25-PH-gfp^ was less than that in the cells with vAc^Pp10-e25-PH-gfp^ (data not shown). Reduction of polyhedrin in the infected cells could affect formation of OBs.

## Materials and Methods

### Virus, cell line and primers

The AcMNPV bacmid bMON14272 was maintained in DH10B cells as described previously [38]. The Sf9 cell line (Invitrogen), a clonal isolate of the parent cell line IPLB-Sf21-AE from the fall armyworm *Spodoptera frugiperda* [39] were cultured at 27°C in Grace's medium supplemented with 10% fetal bovine serum, penicillin and streptomycin.

The DNA primers used in this study were synthesized by GenScript, Inc. and are shown in Table 1.

**Table 1:** Oligonucleotides used to generate knockout and repair bacmid constructs.

Name	Sequence
dhUP	5′CGTGCCTGCAGCTCAGCAAGTTTTATT3′
dhDP	5′CGTAAGCTTGAGCACCAACAATAGTCCG3′
catUP	5′CGCGGATCCTTCGAATAAATACCTGTG3′
catDP	5′ATAACTGCAGCCAGCAATAGACATAAGCG3′
uhUP	5′CGAGTCTAGACGATCGTGCTGTACTTGTGT3′
uhDP	5′GCGCGGATCCTGATTTGTTTTAATTTAC3′
polhUP	5′AAGCGAATTCTAACAGCCATTGTAATGAGACG3′
polhDP	5′CAGCGTATACGCCCGATGTTAAATATGTCC3′
Pgp16UP	5′AGATTCTAGATCATAGCAACGATGCGGCC3′
gfpDP	5′GCGCTCGAGTTACTTGTACAGCTCGTCC3′
e25UP	5′CTAAGGCCTTGTTCGATGCAATGAT3′
e25DP	5′GCGCTCTAGACGAATATAAACGCAAGT3′
e25UP2	5′GTAGGATCCATCATGTGGGGAATCGTGTT3′
e25DP2	5′ACGCGAGCTCGAATATAAACGCAAGTAGT3′
Pie1UP	5′GCCAAGCTTGTACGGCTCGCATGT3′
Pie1DP	5′ATGGGATCCAGTCACTTGGTTGTTCACG3′
Pie1UP2	5′GATAGGCCTGTACGGCTCGCATGT3′
Pp10UP	5′GTTAAGCTTTGGCGACCCAACATGTCC3′
Pp10DP	5′GCGCGGATCCGATTGTAAATAAAATGT3′
Pp10UP2	5′GATAGGCCTTGGCGACCCAACATG3′
Pp10UP3	5′TATATCTAGATGGCGACCCAACATGTCCGT3′
e25UP3	5′GACCGAGCTCTTGTTCGATGCAATGAT3′
e25DP3	5′GCGCTACGTAGAATATAAACGCAAGT3′
gusUP	5′AGTCCTCGAGATGTTACGTCCTGT3′
gusDP	5′AGTCAAGCTTTCATTGTTTGCCTCCCTGC3′
Pvp39UP	5′ACCGTCTAGAATATTGTTGTGCCAGC3′
Pvp39DP	5′CTCGCTCGAGATTGTTGCCGTTATAAATATGG3′
Pie1UP3	5′TATCGAGCTCGTACGGCTCGCATGT3′
polhUP2	5′GAGTCTGCAGCTCGAGTAACAGCCATTGTAAT3′
polhDP2	5′TTAGCATGCTTAATACGCCGGACCAGT3′
polhUP3	5′GCGTTCTAGAGTTCACCTCCCTTTTC3′
polhDP3	5′TAGCTGCAGTTAATACGCCGGACC3′
M13F	5′GTTTTCCCAGTCACGAC3′
M13R	5′CAGGAAACAGCTATGAC3′
e25UP4	5′GCGCTCCATGGATGCATTAAATTTCAATTC3′

Sequences of restriction sites are underlined.

### Construction of *e25* knockout AcMNPV bacmid

The AcMNPV bacmid bMON14272 was used to generate an *e25* KO virus by recombination in *E. coli* using the λ Red system, as previously described [40]. A DNA fragment (DS) corresponding to the 3′-end (nt 80563–80578) of AcMNPV *e25* was amplified by PCR with the primers dhUP and dhDP, and inserted into the PstI and HindIII sites of pUC19. The *cat* was amplified with the primers catUP and catDP and inserted into the BamHI and PstI sites of the resultant plasmid. Then, the *cat*-DS fragment was isolated and inserted into the BamHI and HindIII sites of pBluescript II KS (-); and another DNA fragment (US) corresponding to the upstream sequence of *e25* (nt 79492–79971) was amplified with the primers uhUP and uhDP, and inserted upstream the *cat* gene. The resultant plasmid was cut with XbaI and HindIII to isolate the US-cat-DS segment, which was electro-transformed into arabinose-induced *E. coli* DH10B cells harboring bMON14272 and pKD46 encoding λ-Red recombinase. The resultant bacmid was named vAc^e25ko^ (Fig. 1B). Four sets of primers, uhUP/dhDP, uhUP/catDP, catUP/dhDP and catUP/catDP were used in PCR to confirm the proper replacement of *e25* with *cat* cassette in the bacmid.

### Construction of *e25* knockout, repair, and wt AcMNPV bacmids containing *egfp* and/or *polh*

A DNA fragment containing AcMNPV *polh* with native promoter was PCR-amplified using the primers polhUP and polhDP. It was inserted between the EcoRI and BstZ17I sites of pFastBac1 (Invitrogen) to obtain pFB-PH. Another fragment containing *egfp* under control of an AcMNPV *gp16* promoter was amplified with the primers Pgp16UP and gfpDP, using a plasmid pFB-P_gp16_-gfp (unpublished) as template. It was inserted into the XbaI and XhoI sites of pFB-PH to produce pFB-PH-P_gp16_-gfp. pFB-PH-P_gp16_-gfp was electroporated into *E. coli* DH10B containing bMON14272 and the helper plasmid pMON7124 [38] to generate a *polh*- and *egfp*-containing wt bacmid vAc^PH-gfp^ (Fig. 1A). pFB-PH-P_gp16_-gfp was electroporated into *E. coli* DH10B containing vAc^e25ko^ and pMON7124 to generate a *polh*- and *egfp*-containing *e25*-null bacmid vAc^e25ko-PH-gfp^ (Fig. 1B).

A fragment containing *e25* with the native promoter (nt79729–80868) was amplified using the primer pair e25UP/e25DP. It was inserted into the StuI site of pFB-PH-P_gp16_-gfp to generate pFB-PH-e25-P_gp16_-gfp. Another fragment containing *e25* with the native promoter was amplified using the primers e25UP3 and e25DP3, and inserted between the SacI and SnaBI sites of pFastBac1 to produce pFB-e25. A fragment containing *polh* (nt4300-5257) was amplified with the primer pair polhUP3/polhDP2, and inserted into the XbaI and SphI sites of pFB-e25 to obtain pFB-e25-PH. pFB-PH-e25-P_gp16_-gfp and pFB-e25-PH was electroporated into *E. coli* DH10B containing vAc^e25ko^ and pMON7124 to generate two *e25*-repaired bacmids, vAc^e25ko-rep-PH-gfp^ and vAc^e25ko-rep-PH^ respectively (Fig. 1B).

### Construction of recombinant bacmids with *e25* under control of alternative promoters

A fragment containing *e25* ORF and transcription terminator was amplified with the primers e25UP2/e25DP2, and ligated with pUC19 cut with SacI and BamHI, producing pUC-e25. Another fragment containing an AcMNPV *ie1* promoter (700 bp) was amplified with the primers Pie1UP and Pie1DP, and inserted into the HindIII and BamHI sites of pUC-e25, resulting in pUC-P_ie1_-e25. Using pUC-P_ie1_-e25 as template, a fragment containing the *e25* under control of the *ie1* promoter was amplified with primers Pie1UP2 and e25DP2, and ligated with pFB-PH-P_gp16_-gfp cut with StuI and SacI, to produce pFB-P_ie1_-e25. In the same way, a fragment containing AcMNPV *p10* promoter (297 bp) amplified with the primers Pp10UP and Pp10DP was inserted upstream of the *e25* ORF of pUC-e25 to produce pUC-P_p10_-e25, which was used as template to amplify a fragment containing *e25* linked with a *p10* promoter with the primers Pp10UP2/e25DP2. The PCR fragment was inserted between the StuI and SacI sites of pFB-PH-P_gp16_-gfp to construct pFB-P_p10_-e25.

A fragment containing the *polh* (nt4355-5257) was amplified with the primers polhUP2 and polhDP2, and inserted into the PstI and SphI sites of pFastBac1 to obtain pFB-PH-2. Another fragment containing *e25* under control of *p10* promoter was amplified from pFB-P_p10_-e25 with the primers Pp10UP3 and e25DP2, and inserted between the XbaI and SacI sites of pFB-PH-2 to construct pFB-P_p10_-e25-PH. Using pUC-P_ie1_-e25 as template, a fragment containing *e25* under control of *ie1* promoter was amplified with the primers Pie1UP3 and e25DP3; then inserted between the SacI and SnaBI sites of pFastBac1 to make pFB-P_ie1_-e25-2. Another fragment containing *polh* (nt4300-5257) was amplified with primers polhUP3 and polhDP3 and inserted between the XbaI and PstI sites of pFB-P_ie1_-e25, producing pFB-P_ie1_-e25-PH.

pFB-P_ie1_-e25, pFB-P_p10_-e25, pFB-P_p10_-e25-PH and pFB-P_ie1_-e25-PH were electroporated into *E. coli* DH10B containing vAc^e25ko^ and pMON7124 to generate *e25*-repaired bacmids vAc^Pie1-e25-PH-gfp^, vAc^Pp10-e25-PH-gfp^, vAc^Pie1-e25-PH^ and vAc^Pp10-e25-PH^ respectively (Fig. 1B).

### Construction of recombinant bacmids containing reporter gene under control of *vp39* promoter

Using pFB-PH-e25-P_gp16_-gfp as template, a fragment containing the *e25* was amplified with the primer pair e25UP3/e25DP3, and inserted into the SacI and SnaBI sites of pFastBac1 to obtain pFB-e25. Using the reporter plasmid pCALL4 [41] as template, a fragment containing GUS coding sequence was amplified with the primer pair gusUP/gusDP. It was inserted into the XhoI and HindIII sites of pFB-e25 to obtain pFB-e25-gus. A fragment containing AcMNPV *vp39* promoter (141 bp) was amplified with the primer pairs Pvp39UP/Pvp39DP, and inserted into the XbaI and XhoI sites of pFB-e25-gus to obtain pFB-e25-P_vp39_-gus. The *gus*-containing PCR fragment was inserted into the XhoI and HindIII sites of pFB-P_ie1_-e25-2 to make pFB-P_ie1_-e25-gus. The PCR fragment containing the *vp39* promoter was inserted into the XbaI and XhoI sites of pFB-P_ie1_-e25-gus, producing pFB-P_ie1_-e25-P_vp39_-gus. pFB-P_ie1_-e25-P_vp39_-gus was cut with EcoRI and XbaI to remove *ie1* promoter and most part of the *e25* sequence, then, end-filled and re-circularized, to obtain pFB-P_vp39_-GUS. pFB-e25-P_vp39_-gus, pFB-P_ie1_-e25-P_vp39_-gus and pFB-P_vp39_-GUS were electroporated into *E. coli* DH10B containing vAc^e25ko^ to generate bacmids vAc^e25-Pvp39-gus^, vAc^Pie1-e25-Pvp39-gus^ and vAc^e25ko-Pvp39-gus^ respectively (Fig. 5A).

All the transfer vectors above were sequenced to confirm the construction. All of the bacmid constructs made by transposition were confirmed by PCR with primer set M13F/R according to the manufacturer's protocol.

### Titration of BV

Sf9 cells were transfected with the appropriate bacmids (2.0 µg/well) or infected with infectious cell culture supernatants. The cell culture supernatants were collected at various time points, and the budded virus titers were determined using a TCID_50_ end-point dilution assay [42]. Virus infection was determined by monitoring GFP expression with fluorescence microscopy.

### Quantitative real-time PCR

Viral DNA replication was assayed by real-time PCR, as previously described [43] with modifications. Sf9 cells seeded in 35 mm dishes (1.0×10^6^ cells/dish) were transfected with vAc^e25ko^, vAc^Pie1-e25-PH-gfp^, or vAc^gp64ko^ (5 µg/dish). At designated time points post transfection, medium was removed from selected dishes; the cells were washed twice with PBS (pH 7.4); then, 1 ml of PBS containing 0.5% Triton X-100 was added into each dish. The cells were harvested and the DNA was extracted using a combination of two freeze-thaw cycles, protease, RNase treatment followed by phenol-chloroform-isoamyl alcohol extraction. Finally, the DNA pellet was dissolved in 100 µl of ddH_2_O. Prior to the PCR, 8 µl of total DNA from each time-point was digested with 5 U of DpnI for 20–24 h, in a 20 µl of total reaction volume. An aliquot of the digested DNA (2 µl) was combined with the SYBR-GreenI Real-Time PCR Master Mix Kit (Toyobo) and the qPCR primers Q-65972F and Q-66072R (42) in a 20 µl reaction. The samples were analyzed in a Bio-Rad CFX96 qPCR cycler under the following conditions: 1 cycle of 95°C for 3 min; 40 cycles of 95°C for 10 s, 60°C for 30 s. The results were analyzed using CFX Manager 2.1 (Bio-Rad) software.

### GUS assays

Late gene expression (*gus* under control of the promoter of AcMNPV *vp39*) assays were done following a previous protocol with modifications [44]. Sf9 cells seeded in 96-well plates at a density of 1.0×10^5^ cells/well were transfected with 0.5 µg of the individual designated bacmids, using liposomes. Grace's medium was used to make the transfection mixture. Transfected cells were incubated with the transfection mixture at 26°C for 5 h, after which it was replaced with fresh Grace's medium supplemented with 10% FBS. Cell samples were collected and processed at time points 0, 12, 24, 36, 48 h.p.t., as described below: Medium in dishes was removed, and PBS buffer was added to rinse the cell layer twice. Then, 100 µl of Glo Lysis Buffer (promega) was added to each dish, and incubated for 5 min. The cell lysate was harvested into an eppendorf tube and subjected to centrifugation at 12,000 rpm for 10 min to pellet cell debris. To test GUS activity, 20 µl of the cellular extract was mixed with 80 µl of pre-warmed (37°C) MUG solution (1 mM in ddH_2_O) and incubated at 37°C for 10 min. The reaction was terminated by addition of 400 µl of 0.2 M Na_2_CO_3_. Fluorescence was then measured with excitation at 365 nm, emission at 455 nm on a FLX800 spectrofluorimeter (Biotek). All GUS expression values were derived from three independent transfections.

### Preparation of polyclonal antibodies against E25

A truncated *e25* ORF (nt80028–80657), with the 5′-end sequence encoding a putative trans-membrane domain omitted, was amplified as a NcoI -XhoI fragment with primers e25UP4 and e25DP4, and inserted into the correspondent sites of pPROExHTa (Invitrogen) to construct pPRO-e25t, in which the truncated *e25* was fused with a 5′-tag sequence encoding six histidine residues. The plasmid was transformed into *E. coli* BL21 (DE3) pLysS cells; and the HIS-tagged E25t was purified by using the Ni-NTA resin (Qiagen), following the manufacturer's protocol. A rabbit was injected with 400 µg of the HIS-tagged E25t protein in complete Freund's adjuvant. Two weeks after the first inoculation, the animal was subjected to two boosts of 400 µg at 2-week interval in incomplete Freund's adjuvant. Nine days after the final boost, the animal was bled and the serum was prepared for use in this study.

### Western Blot analysis

Sf9 cells seed in 35 mm plates were transfected with a designated bacmid were harvested at designated time points post transfection. The cell pellets were resuspended individually in 100 µl of PBS and mixed with 25 µl of 5X loading buffer (60 mM Tris-HCl, pH 6.8, 2% SDS, 25% glycerol, 14.4 mM β-mercaptoethanol, 0.1% bromophenol blue), then incubated at 100°C for 5 min. The cell lysate was centrifuged at 12,000 rpm for 5 min. The protein samples (supernatant) were separated by SDS–12% polyacrylamide gel electrophoresis (PAGE) and transferred to BioTace PVDF membrane (PALL Life Science) with a liquid transfer apparatuses. The blots were probed with the AcMNPV E25-specific rabbit antiserum prepared above. IRDye-800CW conjugated goat anti-rabbit antibody (1∶10,000) (LI-COR) was used as the secondary antibody. Fluorescence was detected by LI-COR Odyssey. SDS-PAGE and immunohybridizations to western blots were performed in accordance with standard protocols and manufacturer's instruction [45].

### Immuno-fluorescence assays and confocal microscopy

To perform immuno-fluorescence assays, Sf9 cells were seeded on the surface of coverslips placed in 35 mm dishes at 2×10^5^ cells/dish and incubated overnight. Then the cells were inoculated with infectious supernatant of AcMNPV at a MOI of 5, or, transfected with wt and recombinant bacmids respectively. At 48 h.p.t., or designated time points after infection, the cells on the coverslips were fixed with immunol staining fix solution (Beyotime), incubated with E25-specific antibody, then, incubated with Rhodamine (TRITC)-conjugated goat-anti-rabbit IgG (PTG Lab) (1∶60) and stained with Hoechst33258 (Beyotime) sequentially, following standard methods or manufacturer's recommendation. Finally, the cells were sealed on microscope slides with antifade mounting medium (Beyotime), and subjected to a confocal microscopic assay with a ZEISS, LSM710 NLO confocal laser scanning microscope for fluorescence using a wavelength of 488 nm laser line for GFP, 550 nm for Rhodamine, and 352 nm for Hoechst33258. All images were digitally recorded and merged by the use of ZEISS software.

### Electron microscopy

For electron microscopy, 1×10^6^ Sf9 cells per dish (35 mm) were transfected with 1.0 µg of vAc^e25ko-PH^, vAc^Pie1-e25-PH^, vAc^Pp10-e25-PH^ or vAc^e25ko-rep-PH^. At 72 and 96 h.p.t., cells were fixed, dehydrated, then dislodged with a rubber policeman and precipitated by centrifuge at 3,000 rpm for 5 min. The cell pellets were embedded, sectioned, and stained as described previously [46], then examined with a FEI Tecnai G^2^ 20 TWIN transmission electron microscope at an accelerating voltage of 200 kV.
